# Bombesin-like receptor 3 (*Brs3*) expression in glutamatergic, but not GABAergic, neurons is required for regulation of energy metabolism

**DOI:** 10.1016/j.molmet.2017.08.013

**Published:** 2017-09-15

**Authors:** Cuiying Xiao, Ramón A. Piñol, Jesse Lea Carlin, Cuiling Li, Chuxia Deng, Oksana Gavrilova, Marc L. Reitman

**Affiliations:** 1 Diabetes, Endocrinology, and Obesity Branch, National Institute of Diabetes and Digestive and Kidney Diseases, NIH, Bethesda, MD 20892, USA; 2 Genetics of Development and Disease Branch, National Institute of Diabetes and Digestive and Kidney Diseases, NIH, Bethesda, MD 20892, USA; 3 Faculty of Health Sciences, University of Macau, Macau SAR, China; 4 Mouse Metabolism Core, National Institute of Diabetes and Digestive and Kidney Diseases, NIH, Bethesda, MD 20892, USA

**Keywords:** Bombesin-like receptor 3, Obesity, Energy expenditure, Food intake, Glutamatergic neurons, Body temperature

## Abstract

**Objective:**

Bombesin-like receptor 3 (BRS-3) is an orphan G protein-coupled receptor. *Brs3* null mice have reduced resting metabolic rate and body temperature, increased food intake, and obesity. Here we study the role of *Brs3* in different neuron types.

**Methods:**

Mice able to undergo Cre recombinase-dependent inactivation or re-expression of *Brs3* were generated, respectively *Brs3*^*fl/y*^ and *Brs3*^*loxTB/y*^. We then studied four groups of mice with *Brs3* selectively inactivated or re-expressed in cells expressing *Vglut2*-Cre or *Vgat*-Cre.

**Results:**

Deletion of *Brs3* in glutamatergic neurons expressing Vglut2 reproduced the global null phenotype for regulation of food intake, metabolic rate, body temperature, adiposity, and insulin resistance. These mice also no longer responded to a BRS-3 agonist, MK-5046. In contrast, deletion of *Brs3* in GABAergic neurons produced no detectable phenotype. Conversely, the wild type phenotype was restored by selective re-expression of *Brs3* in glutamatergic neurons, with no normalization achieved by re-expressing *Brs3* in GABAergic neurons.

**Conclusions:**

*Brs3* expression in glutamatergic neurons is both necessary and sufficient for full *Brs3* function in energy metabolism. In these experiments, no function was identified for *Brs3* in GABAergic neurons. The data suggest that the anti-obesity pharmacologic actions of BRS-3 agonists occur via agonism of receptors on glutamatergic neurons.

## Highlights

• *Brs3* in glutamatergic neurons regulates food intake, metabolic rate, and body weight.
• *Brs3* in glutamatergic neurons is both necessary and sufficient for these functions.
• No phenotypes were identified by *Brs3* loss or re-expression in GABAergic neurons.
• BRS-3 agonists likely act on glutamatergic neurons for their anti-obesity effects.

## 1 Introduction

Obesity is an increasing world-wide problem with a profound effect on human health [1], [2]. It is caused by an imbalance between energy intake and energy expenditure, which is regulated by the brain. Within the brain, the hypothalamus has a major role, integrating endocrine and peripheral nerve signals with inputs from other brain regions and sending signals centrally and peripherally.

Bombesin-like receptor 3 (BRS-3) is an orphan G protein-coupled receptor (GPCR), first identified via sequence similarity to the gastrin releasing peptide and neuromedin B receptors [3], [4], [5]. To date, attempts to identify a mammalian endogenous BRS-3 ligand have been unsuccessful [6], [7], [8], [9], although in chicken and spotted gar (a fish), gastrin releasing peptide and neuromedin B both appear to be endogenous ligands [10]. Mammalian BRS-3 may be conserved during evolution because it can modify the response pattern of other GPCRs or contribute to basal signaling [11]. *Brs3* mRNA and BRS-3 binding activity are located in restricted regions of the brain, including portions of the hypothalamus, amygdala, and thalamus [12], [13], [14], [15], [16], [17], [18], [19]. BRS-3 is also reported to be present at low levels in peripheral sites including pancreatic islets, developing testis, female reproductive tract, lung, and muscle, and in certain cancers [5], [20], [21], [22] (https://gtexportal.org/home/gene/BRS3).

Insights into the function of BRS-3 were hugely advanced by development of a *Brs3* knockout (KO) mouse [23]. The null phenotype includes obesity, increased food intake and meal size, and reductions in metabolic rate, resting body temperature, and resting heart rate [23], [24], [25], [26], [27]. The development of potent and selective ligands has expanded our knowledge of BRS-3 ([15], reviewed in [28], [29]). Treatment with a BRS-3 antagonist increased food intake and body weight, while agonists reduced food intake, increased resting metabolic rate, body temperature, and heart rate [15], [27], [30], [31]. One BRS-3 agonist, MK-5046, reached initial human studies but increased blood pressure and was discontinued [32]. It is currently unknown which cells mediate these functions of BRS-3. For example, it has not even been established if neuronal BRS-3 are the relevant receptors for regulation of body weight and energy homeostasis.

Conditional deletion removes gene function in precisely defined subsets of cells, supplying evidence for the necessity of the gene (e.g., [33], [34]). Conversely, reconstitution by conditional re-expression identifies those cells that are sufficient for gene function. For example, these tools have been used to elucidate the hypothalamic MC4R neurons that regulate food intake [35].

To dissect the functions of *Brs3*, we developed mice that allow selective, conditional deletion or re-expression of *Brs3*. We use these mice to investigate the necessity and sufficiency of *Brs3* in glutamatergic and GABAergic neurons for regulation of energy homeostasis and identify a role for *Brs3* in glutamatergic but not GABAergic neurons.

## 2 Experimental methods

### 2.1 Mice and reagents

All mice were given ad libitum access to water and chow (7022 NIH-07 diet, 15% kcal fat, energy density 3.1 kcal/g, Envigo Inc., Indianapolis, IN) or high fat diet (HFD, D12492, 60% kcal fat, 5.24 metabolizable kcal/g; Research Diets, New Brunswick, NJ) with a 12:12-h dark–light cycle (lights on at 0600 h) and Teklad bedding (either 7090 or TEK-Fresh, Envigo Inc.). Protocols were approved by the NIDDK Animal Care and Use Committee. MK-5046 [36] (vehicle: 10% Tween 80 in 0.25% methylcellulose) was generously provided by Merck Research Laboratories (Rahway, NJ). MTII (vehicle: saline) was purchased from Bachem (Torrance, CA).

*Brs3* is located on the X chromosome, so all experiments were performed in male mice. *Brs3*^*-/y*^ mice were provided by Dr. James Battey [24] and back-crossed at least eight generations onto a C57BL/6J background. Vglut2-ires-Cre (JAX 028863 [34], hereafter *Vglut2-Cre*) and Vgat-ires-Cre (JAX 028862 [34], hereafter *Vgat-Cre*) mice were supplied by Dr. Michael Krashes, NIDDK. *Brs3* inactivation studies used littermate male progeny of female *Brs3*^*fl/fl*^ × male *Brs3*^*+/y*^*;Cre/+* matings, on a mixed background (129SvEv, Black Swiss, and C57BL/6J). *Brs3* re-expression studies used littermate male progeny of female *Brs3*^*loxTB/loxTB*^ × male *Brs3*^*+/y*^*;Cre/+*, female *Brs3*^*loxTB/ko*^ × male *Brs3*^*+/y*^, female *Brs3*^*ko/+*^ × male *Brs3*^*+/y*^, and female *Brs3*^*loxTB/+*^ × male *Brs3*^*+/y*^ mice, on a mixed background. Cohorts of loxTB-*Brs3* mice were studied at different times in the same vivarium, with data pooled for analysis.

*Generation of Brs3 conditional allele, Brs3*^*fl*^. A targeting construct containing a 4.3 kb 5′ arm (SpeI-EcoRV, with a loxP linker in the EcoRI site) and a 3.93 kb 3′ arm (EcoRV-KpnI) was cloned into pLoxPneo1 [37]. TC1 ES cells derived from 129SvEv mice [38] were transfected with linearized construct and selected with G418 and FIAU [37]. Southern hybridization of ES DNA digested with EcoRV using a 5′ flanking probe, detected bands of 5.4 kb (WT) and 3.8 kb (floxed). Targeted ES cells were injected into blastocysts, the resulting chimeric mice were mated with *EIIa-Cre* females (JAX 003314), and a founder having the floxed targeted allele with the selection cassette removed was selected. PCR genotyping used primers x213 (5′-GTATGCATTACCACGTACGA, intron 1, forward) and x214 (5′-GCATTGTCATTCCCAGAGAAA, intron 1, reverse), producing products of 203 bp wild type (WT) and 237 bp (floxed). *Brs3* mRNA was quantitated by RT-PCR using primers x573 (5′-CTGCTGACTTGTGTGCCTGT) and x574 (5′-AGTGGCTTCACGACTGCTTT).

*Generation of Brs3 conditional re-expression allele, Brs3*^*loxTB*^. This strategy is based on the loxTB MC4R [35] and loxTB MC3R [39] mice. For those single-exon genes, the loxTB was placed upstream of the start codon. We situated the same loxTB cassette 421 bp into the first intron of *Brs3* to produce the loxTB-*Brs3* mice (inGenious Targeting Laboratory, Ronkonkoma, NY). A C57BL/6 BAC clone (RP23-378L4) was used to generate the targeting construct with a 6.05 kb 5′ arm, the 2924 bp loxTB cassette ([35] provided by Dr. Joel Elmquist, University of Texas Southwestern Medical Center), and a 2.15 kb 3′ arm. Correctly targeted iTL IC1 (C57BL/6) embryonic stem cells were microinjected into Balb/c blastocysts and the resulting chimeras were bred with C57BL/6N mice. PCR genotyping with x559 (5′-TGCAGGTGCAAAGAAAAATG, forward, upstream of loxTB), x561 (5′-GCGCCAGAACATTTCTATCC, forward, within loxTB), and x562 (5′-CCAGGGAGCTGAAAACCTTA, reverse, downstream of loxTB), generates a 214 bp product (x559–x562) with WT *Brs3*, a 248 bp product (x559–x562) with Cre-recombined loxTB, and a 395 bp product (x561–x562) with unrecombined loxTB (the possible 3165 bp product of x559–x562 is not successfully amplified). loxTB recombination was quantitated by PCR using primers x669 (5′- GGACTCTGCACCATAACACAC) and x212 (5′- ACGGTCCATTTCCCACCTAT).

### 2.2 Phenotyping

Body weight and food intake were measured weekly. Body composition was measured by time domain Echo MRI 3-in-1 (Echo Medical Systems, Houston, TX) every two weeks. Energy expenditure was estimated by energy balance of singly-housed mice in their home cage environment. In brief, estimated energy expenditure is calculated from the metabolizable caloric intake, corrected for the change in caloric content of the mouse (from the change in body composition over the measurement interval) [40].

*Glucose and insulin tolerance tests, hormone and metabolite profiles*: Intraperitoneal glucose (2 g/kg for chow-fed mice, 1 g/kg for HFD-fed mice, with AUC calculated from 0 mg/dl) tolerance tests were performed at 0900, following an overnight (16 h) fast. Glucose was measured with a Glucometer Contour (Bayer, Mishawaka, IN). Insulin (0.75 unit/kg, i.p.) tolerance tests were performed at 0900, in non-fasted mice, with AUC calculated from 0 mg/dl. Blood was collected at 0900 by tail bleed at 20 weeks of age for fed glucose and serum insulin, free fatty acid, cholesterol, and triglycerides measurement. Serum for other measurements was taken from anesthetized (100 mg/kg ketamine and 10 mg/kg xylazine, ip) mice by retro-orbital bleed at euthanasia and frozen until assayed. Free fatty acids (FFA, Roche Diagnostics GmbH, Mannheim, Germany), triglycerides (Pointe Scientific Inc., Canton, MI), and cholesterol (Thermo Scientific, Middletown, VA) were measured using the indicated colorimetric assays. Leptin (R&D Systems, Minneapolis, MN) and insulin (Crystal Chem, Downers Grove, IL) were measured by ELISA.

*Treatment effect on food intake and energy expenditure*: Food intake was performed as described [15]. In brief, mice were fasted for 4 h, dosed with drug or vehicle 30 min before lights off, and access to chow was provided at lights out. Food intake over the next 2 h was measured. Energy expenditure at thermoneutrality was measured as described [15]. The ratio of post-dosing (120–240 min) to pre-dosing (−150 to −30 min) energy expenditure was calculated.

*Telemetric monitoring of body temperature:* Core body temperature (Tb) and activity were continuously measured by telemetry using G2 E-mitters implanted intraperitoneally, ER4000 energizer/receivers, and VitalView software (Starr Life Sciences, Oakmont, PA) with data collected each minute. Only data collected at the same time were compared, to control for variation in environmental temperature, noise, and disturbances in the vivarium. We re-analyzed prior data [26] to identify a metric that distinguishes the WT and *Brs3* null phenotypes. Using 24-hour (or multiples thereof) datasets with Tb measured every minute, the range (defined as the difference between the 95th and 5th percentiles) and the standard deviation were both sensitive and robust in discriminating WT and *Brs3* null mice (Table S1). These metrics intrinsically minimize the effect of Tb accuracy errors, such as due to telemeter placement or calibration. The metric was not improved by selective analysis of light vs dark phase and/or active vs resting intervals.

*Quantitative PCR and RT-PCR*: Tissue DNA and RNA were extracted (Qiagen Allprep DNA/RNA micro Kit, Germantown, MD). RNA was reverse transcribed (Roche Transcriptor High Fidelity cDNA Synthesis Kit, Indianapolis, IN). DNA and cDNA were quantified by real-time polymerase chain reaction (q-PCR, Applied Biosystems 7900HT, Foster City, CA) using SYBR green, normalized to 18S RNA.

*Experimental design and statistical analysis*: Data are presented as mean ± SEM, and were analyzed by t-test, one-way, or two-way repeated measures ANOVA with Holm-Sidak post-hoc testing, using Sigmaplot 12.5 (Systat Software Inc) or Prism v7.03 (GraphPad). Statistical significance was defined as 2-tailed P < 0.05.

## 3 Results

### 3.1 Generation of *Brs3*^*fl/y*^ mice

**Figure 1:**
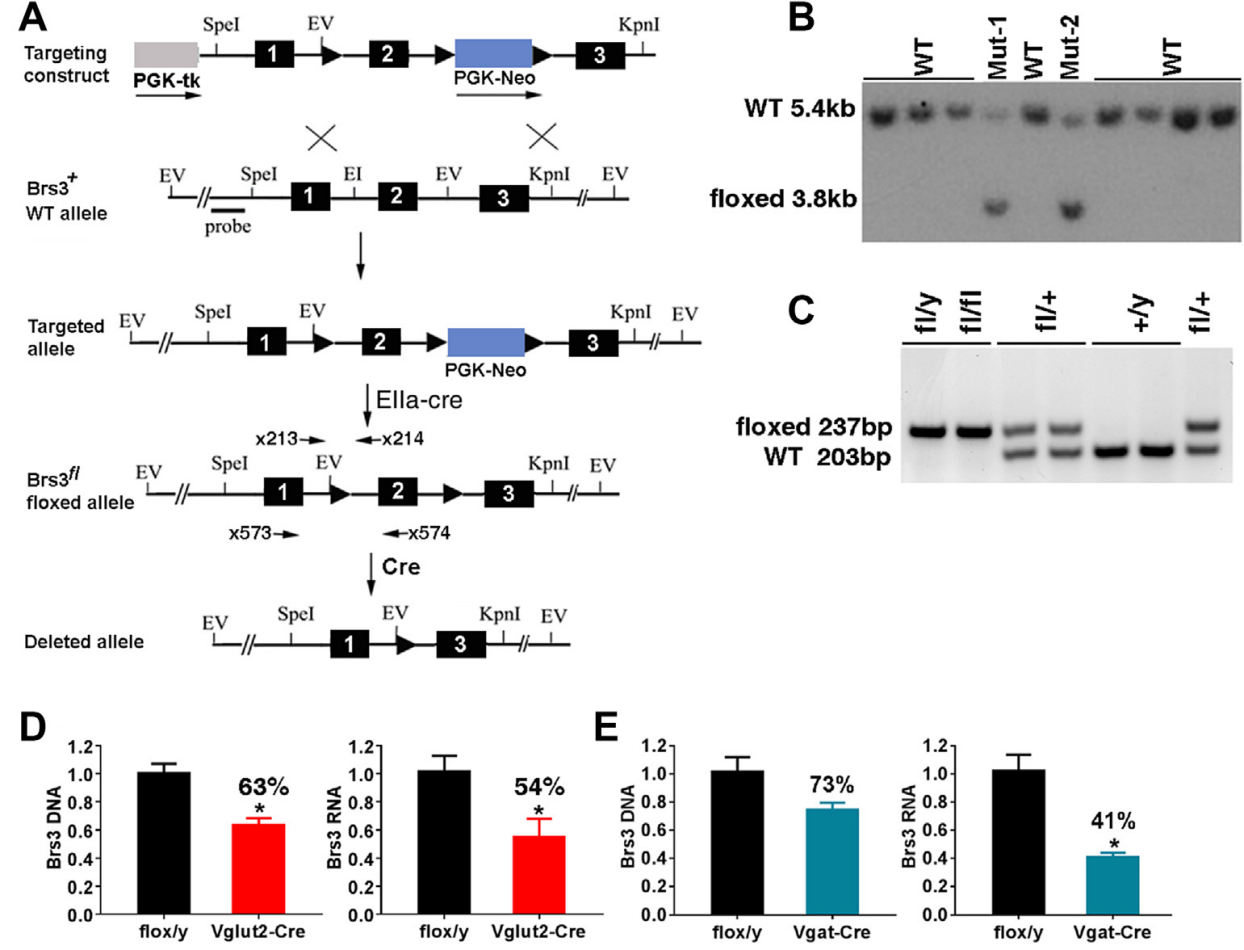
Generation of *Brs3*^*fl/y*^ mice. (A) Scheme for creation of floxed *Brs3* allele. Homologous recombination of the *Brs3* targeting construct in embryonic stem (ES) cells produced the targeted allele, and a founder was bred with an EIIa-Cre female to remove the PGK-Neo cassette. Deletion of exon 2 by Cre recombinase generates the null allele. (B) Southern hybridization of ES DNA digested with EcoRV using a 5′ flanking probe, detected bands of 5.4 kb (WT) and 3.8 kb (floxed). (C) PCR genotyping using primers x213 and x214, produces products of 203 bp (WT) and 237 bp (floxed). (D, E) Brs3 DNA and RNA were quantified by PCR in hypothalamus of (D) *Brs3*^*fl/y*^*;Vglut2-Cre* (Vglut2-Cre) or (E) *Brs3*^*fl/y*^*;Vgat-Cre* (Vgat-Cre) and littermate control *Brs3*^*fl/y*^ (flox/y) mice. Primers x213 and x214 were used for DNA and x573 and x574 for RNA. N = 4–5/group; * indicates P < 0.05 by unpaired t-test.

To enable conditional deletion of *Brs3*, we generated a floxed allele (*Brs3*^*fl*^) by placing Cre recombinase recognition loxP sites flanking exon 2, which is required for *Brs3* function [23], [24], [25] (Figure 1A–C). Insertion of loxP sites had no effect on body weight (not shown), indicating that these sequences are not deleterious.

### 3.2 Selective inactivation of *Brs3* by *Vglut2-Cre* reproduces the global knockout phenotype

We hypothesized that neuronal BRS-3 was responsible for regulation of body weight and energy homeostasis and chose to gain more information than a pan-neuronal knockout would provide. Since *Brs3* is expressed in both glutamatergic [17] and GABAergic ([41] and unpublished observations) neurons, we used Brs3^fl/y^ mice to examine the effects of germline loss of Brs3 in glutamatergic neurons expressing *Vglut2* or in GABAergic neurons expressing *Vgat*. The level of *Brs3* deletion was quantified in hypothalamic tissue. In *Brs3*^*fl/y*^*;Vglut2-Cre* mice, we detected 37% lower *Brs3* DNA levels and 46% lower *Brs3* RNA levels (Figure 1D). In *Brs3*^*fl/y*^*;Vgat-Cre* mice, there was 27% reduction of *Brs3* DNA and 59% reduction in *Brs3* mRNA levels (Figure 1E). Thus, Cre recombinase successfully deleted the floxed *Brs3* exon, indicating that *Brs3* is expressed in *Vglut2* and *Vgat* neurons in the hypothalamus.

**Figure 2:**
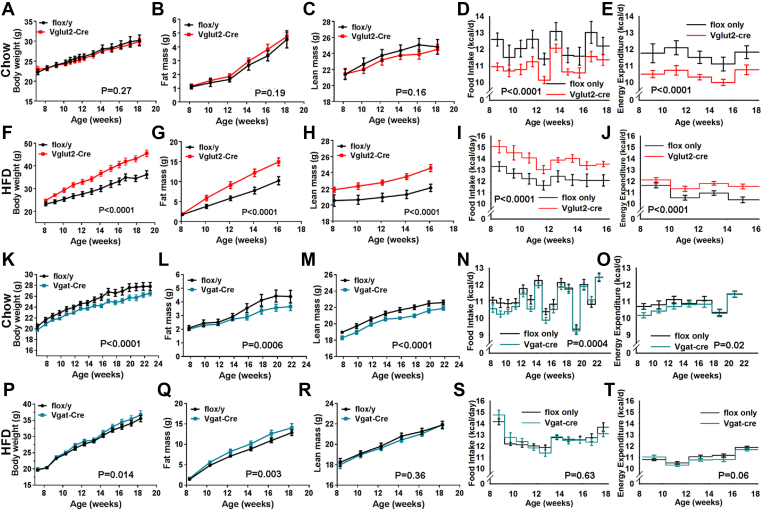
Effect of *Brs3* deletion on body weight, composition, and energy homeostasis. Mice with *Brs3* deleted in *Vglut2-* or *Vgat*-expressing neurons were fed a chow or high fat diet (HFD). (A–E) *Brs3*^*fl/y*^*;Vglut2-Cre* (Vglut2-Cre) and littermate control *Brs3*^*fl/y*^ (flox/y) mice on chow (N = 6/group). (F–J) the same genotypes on HFD (N = 9/group). (K–O) *Brs3*^*fl/y*^*;Vgat-Cre* (Vgat-Cre) and littermate control *Brs3*^*fl/y*^ (flox/y) mice on chow (N = 10–13/group). (P–T) the same genotypes on HFD (N = 11–13/group). P values were determined by 2-way RM ANOVA.

On a chow diet, body weight, fat mass, and lean mass of *Brs3*^*fl/y*^*;Vglut2-Cre* mice were remarkably similar to that of the *Brs3*^*fl/y*^ control mice (Figure 2A–C, Figure S1A–C). However, the matched body weight and composition were achieved with a 9.9% lower food intake and a 10.3% lower average metabolic rate over 10 weeks (Figure 2D,E). On a HFD, *Brs3*^*fl/y*^*;Vglut2-Cre* mice developed obesity, with increased body weight and fat mass, having a 13% increase in food intake (Figure 2F–I). Expressed per mouse, the metabolic rate appeared slightly (7.7%) greater than in the controls (Figure 2J); however, if higher body weight is considered, the metabolic rate was lower than seen in control mice of matched body composition (not shown). Consistent with their increased fat mass, *Brs3*^*fl/y*^*;Vglut2-Cre* HFD-fed mice had heavier BAT, iWAT, and liver, and increased body length (Figure S1D–F).

**Figure 3:**
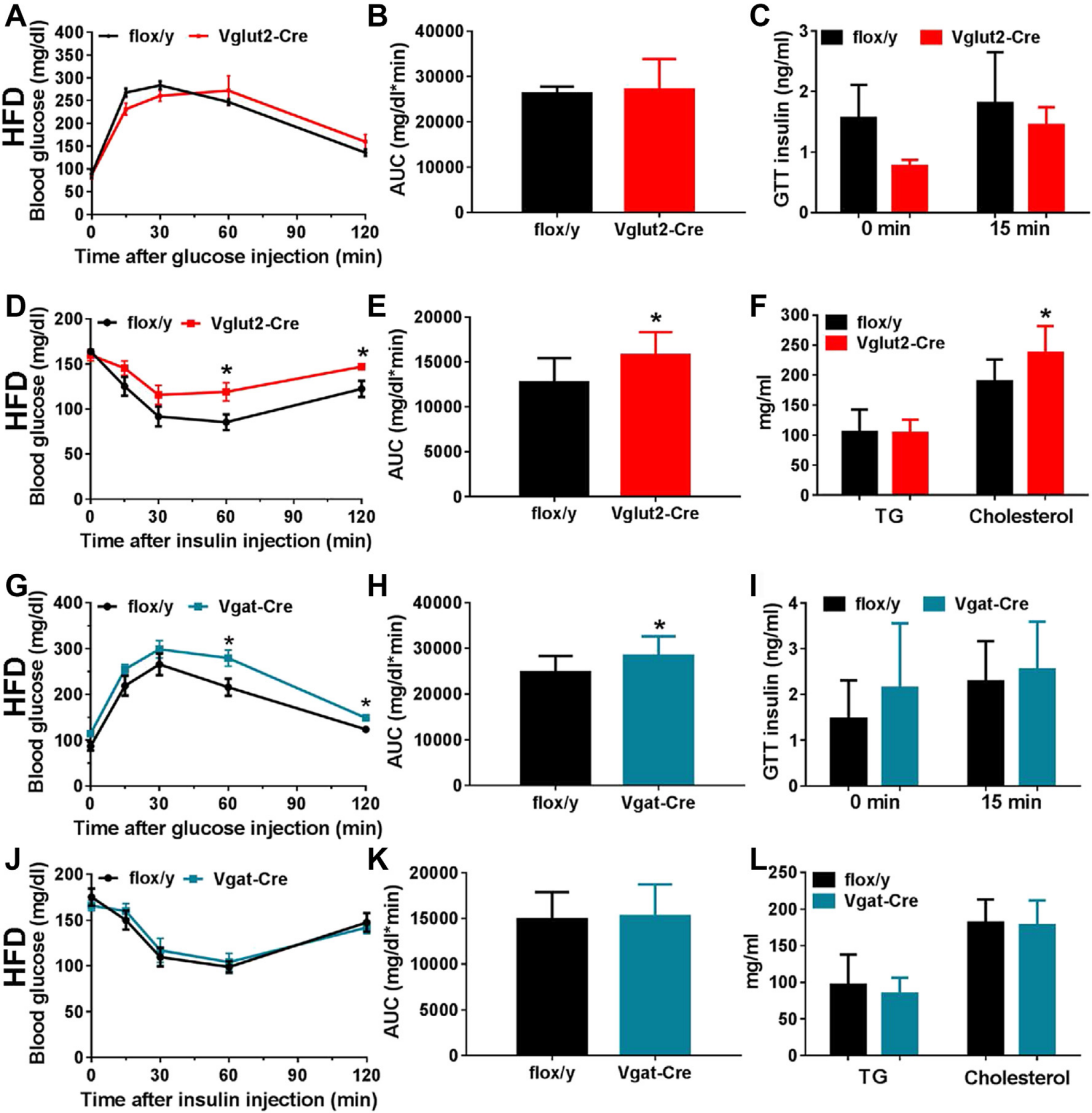
Glucose and lipid metabolism in mice with *Brs3* deleted in *Vglut2-* or *Vgat-*expressing neurons and fed a high fat diet. (A–F) *Brs3*^*fl/y*^*;Vglut2-Cre* (Vglut2-Cre) and littermate control *Brs3*^*fl/y*^ (flox/y) mice. (A–C) Glucose tolerance test, area under the curve (AUC), and insulin at indicated times after glucose challenge in mice at 17 weeks of age. (D, E) Insulin tolerance test and AUC at 19 weeks of age. (F) Serum triglyceride (TG) and cholesterol levels (N = 9/group). (G–L) *Brs3*^*fl/y*^*;Vgat-Cre* (Vgat-Cre) and littermate control *Brs3*^*fl/y*^ (flox/y) mice. (G–I) Glucose tolerance test, area under the curve (AUC), and insulin at indicated times after glucose challenge. (J, K) Insulin tolerance test and AUC. (L) Serum triglyceride (TG) and cholesterol levels (N = 11–13/group). * indicates P < 0.05 by unpaired t-test.

An insulin tolerance test showed insulin resistance, although the glucose tolerance was not different from control mice (Figure 3A–E). Serum cholesterol was increased 25% and triglycerides levels were unchanged (Figure 3F).

Mild behavioral phenotypes have been reported in *Brs3*^*-/y*^ mice, including a greater preference for saccharine and aversion to quinine [13], increased meal size [24], less anxiety in an elevated plus maze test and decreased response to social isolation [42]. We measured preferences for sucrose, saccharin, and quinine, but did not detect a clear difference between reference *Brs3*^*-/y*^ and control mice (Figure S2A–C). The *Brs3*^*-/y*^ mice may have a slightly less strong preference for high fat diet over chow (Figure S2D). *Brs3*^*-/y*^ and control mice learned similarly that treats would be presented for a limited time each day (Figure S2E and F). In anxiety assays (open field and elevated plus maze), no phenotype was detected in *Brs3*^*-/y*^ mice (Figure S2G–J). We concluded that any differences observed in *Brs3*^*-/y*^ mice with these assays were not robust enough to warrant testing mice with selective *Brs3* inactivation.

**Figure 4:**
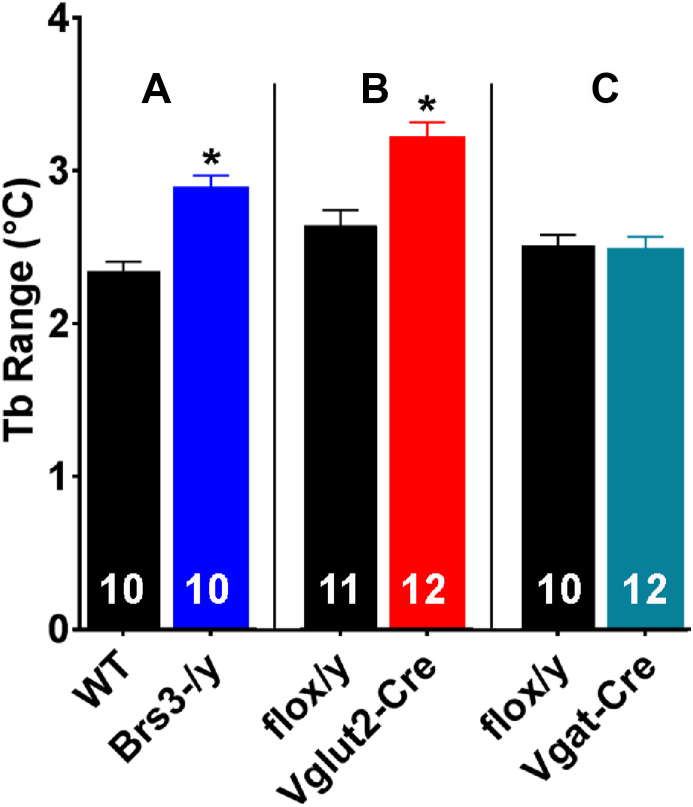
Effect of genotype on core body temperature. Tb was measured by telemetry, sampling for 24 h in singly-housed, free-ranging mice in their home cage. Ambient temperature was ∼22 °C and each comparison is between groups studied simultaneously. We define the Tb range as the difference between the 95th and 5th percentiles. (A) Re-analysis of Tb in WT and global *Brs3*^*-/y*^ mice from [26] showed a 0.54 °C larger range in the global *Brs3*^*-/y*^ mice compared to controls (p = 0.00008). (B) *Brs3*^*fl/y*^*;Vglut2-Cre* (Vglut2-Cre) mice had a 0.59 °C larger range than littermate *Brs3*^*fl/y*^ (flox/y) mice (p = 0.008). (C) *Brs3*^*fl/y*^*;Vgat-Cre* (Vgat-Cre) and littermate *Brs3*^*fl/y*^ (flox/y) mice showed no difference in Tb range (p = 0.78). Additional analyses of the Tb phenotype are in Table S1.

*Brs3*^*-/y*^ mice have a lower core body temperature (Tb) during resting periods in the light phase, but their Tb is comparable to WT mice during active intervals in the dark phase [26]. The 24-hour mean Tb of *Brs3*^*-/y*^ mice is 0.2–0.3 °C below controls, which groups of 6–12 are typically not powered to detect (see [23], [26] and Table S1). However, the *Brs3*^*-/y*^ Tb phenotype is robustly captured as an increase in the range (defined as the difference between the 95th and 5th percentiles) or an increase in the standard deviation of continuous 24-hour body temperature data (Figure 4A, Table S1, Methods 2.2). *Brs3* deletion in *Vglut2-Cre* neurons caused the null phenotype (Figure 4B).

### 3.3 *Brs3* deletion by *Vgat-Cre* does not affect adiposity or body temperature

Unlike the heavier *Brs3*^*fl/y*^*;Vglut2-Cre* mice, the HFD *Brs3*^*fl/y*^*;Vgat-Cre* mice had body weights and fat and lean masses that were near to that of controls, with similar food intake and metabolic rate (Figure 2P–T, Figure S1J–L). *Brs3*^*fl/y*^*;Vgat-Cre* mice on a HFD showed a slight worsening of glucose tolerance (Figure 3G–I), but no difference from controls in insulin tolerance (Figure 3J–K), serum cholesterol and triglyceride levels (Figure 3L), or tissue adiposity (Figure S1J–L). On a chow diet, *Brs3*^*fl/y*^*;Vgat-Cre* mice were slightly leaner than controls at 14–24 weeks of age, but were not different at 52 weeks (Figure 2K–O, Figure S1G–I). *Brs3* deletion in Vgat-Cre neurons had no effect on Tb (Figure 4C). These data suggest that *Brs3* in *Vgat* neurons makes little contribution to the food intake, energy expenditure, Tb, and body weight phenotypes observed in the global *Brs3* null mice.

### 3.4 Loss of response to BRS-3 agonist in *Brs3*^*fl/y*^*;Vglut2-Cre,* but not *Brs3*^*fl/y*^*;Vgat-Cre,* mice

**Figure 5:**
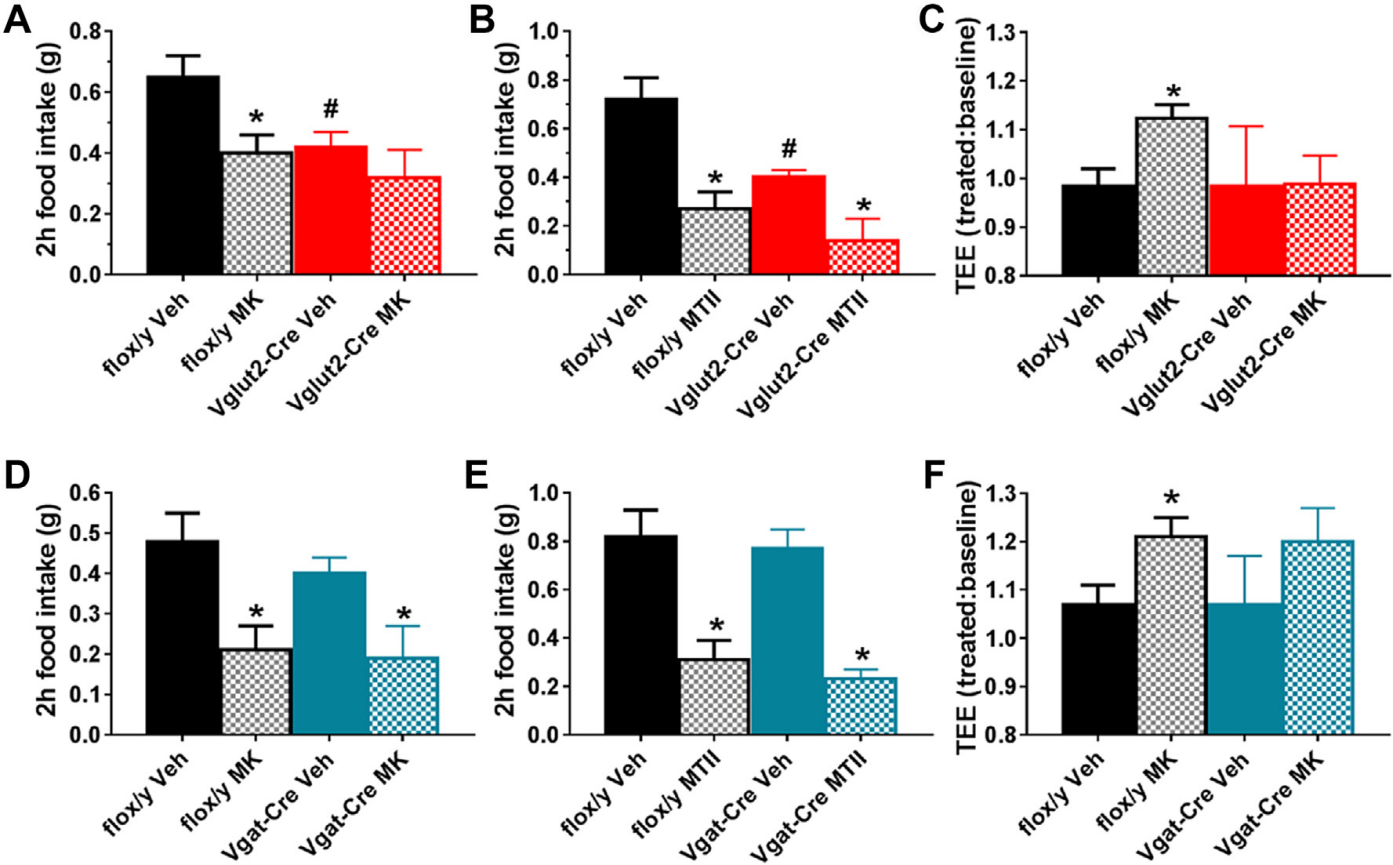
Effect of MK-5046 and MTII on food intake and energy expenditure. (A–C) *Brs3*^*fl/y*^*;Vglut2-Cre* (Vglut2-Cre) and littermate control *Brs3*^*fl/y*^ (flox/y) mice. (A) Effect of MK-5046 (10 mg/kg, i.p.) or vehicle on 2-h food intake. (B) Effect of MTII (3 mg/kg, i.p.) or vehicle on 2-h food intake. (C) Effect of MK-5046 or vehicle on energy expenditure (N = 6/group). Treatment is compared to pre-injection baseline. (D–F) *Brs3*^*fl/y*^*;Vgat-Cre* (Vgat-Cre) and littermate control *Brs3*^*fl/y*^ (flox/y) mice were studied exactly as described in (A–C) (N = 10–12/group for food intake studies, 6/group for energy expenditure study). * indicates P < 0.05 drug vs vehicle, within genotype by paired t-test. # indicates P < 0.05 vehicle between genotypes by unpaired t-test.

The response to the BRS-3 agonist MK-5046 was studied in the chow-fed *Brs3*^*fl/y*^*;Vglut2-Cre* and *Brs3*^*fl/y*^*;Vgat-Cre* mice. The *Brs3*^*fl/y*^*;Vglut2-Cre* mice were heavier than the controls and ate less in the acute food intake assay (Figure 5A,B). MK-5046 reduced food intake in control mice and had no effect in *Brs3*^*fl/y*^*;Vglut2-Cre* mice (Figure 5A). In contrast, the melanocortin agonist MTII reduced food intake similarly in the two cohorts (63% vs 65%) (Figure 5B). Importantly, MK-5046 increased energy expenditure in control, but not in *Brs3*^*fl/y*^*;Vglut2-Cre* mice (Figure 5C). In *Brs3*^*fl/y*^*;Vgat-Cre* mice, both MK-5046 and MTII reduced food intake similarly to their effects in controls (Figure 5D,E). MK-5046 increased energy expenditure with a similar magnitude as in controls, although this was not statistically significant (Figure 5F).

Taken together, these data demonstrate that *Brs3* expression in Vglut2, but not Vgat, neurons is necessary for proper regulation of food intake, metabolic rate, adiposity, adiposity sequelae (insulin resistance), and response to BRS-3 agonist.

### 3.5 Generation of *Brs3*^*loxTB/y*^ mice

**Figure 6:**
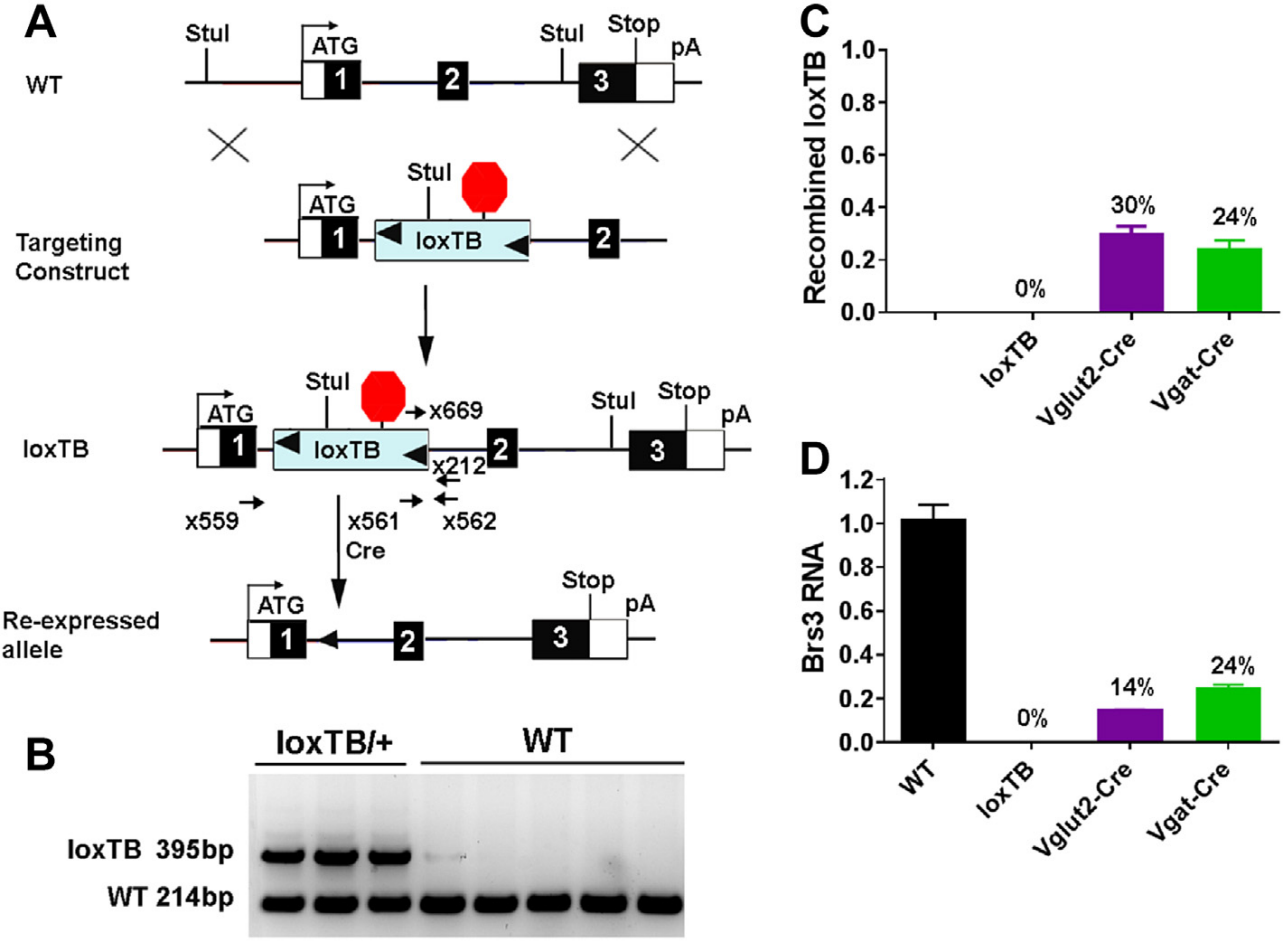
Generation of *Brs3*^*loxTB/y*^ mice. (A) A loxP-flanked transcriptional blocker (loxTB) was inserted in the first intron of *Brs3*. Cre recombinase will cause re-expression of *Brs3* from the silent *Brs3*^*loxTB*^ allele. (B) PCR (primers x559, x561, and x562) genotyping gives 214 bp wild type (WT) and 395 bp unrecombined loxTB products. (C) The fraction of recombined loxTB DNA was measured by subtracting the unrecombined level in the Cre-expressing mice from that in loxTB mice. PCR (primers x669 and x212) was performed on hypothalamus DNA from *Brs3*^*loxTB/y*^ (loxTB), *Brs3*^*loxTB/y*^*;Vglut2-Cre* (Vglut2-Cre), and *Brs3*^*loxTB/y*^*;Vgat-Cre* (Vgat-Cre) mice. (D) *Brs3* RNA in hypothalamus was quantified by RT-PCR (using primers x573 and x574). N = 4–5/group.

We next investigated if selective *Brs3* re-expression was sufficient to reverse the phenotype of global *Brs3* null mice. To do this, we generated a *Brs3* allele with a floxed transcription-blocking sequence, which is removed by Cre recombinase to re-express *Brs3* (Figure 6A,B). To study the *Brs3* re-expression, we generated five genotypes: the new allele (*Brs3*^*loxTB/y*^), alone and with *Vglut2-Cre* or *Vgat-Cre*, WT control (*Brs3*^*+/y*^), and a previously characterized *Brs3* KO (*Brs3*^*-/y*^) [24].

### 3.6 Selective re-expression of *Brs3*

Successful re-expression of *Brs3* was confirmed in hypothalamic tissue. In *Brs3*^*loxTB/y*^*;Vglut2-Cre* mice, 30% recombination of *Brs3* alleles occurred (Figure 6C). This caused re-expression of *Brs3* mRNA to 14% of the level in WT mice (Figure 6D). In *Brs3*^*loxTB/y*^*;Vgat-Cre* mice, there was 24% recombination, producing re-expression at 24% of total WT *Brs3* mRNA levels. No *Brs3* mRNA was detected in *Brs3*^*loxTB/y*^ mice in the absence of Cre recombinase. These results confirm that the loxTB-*Brs3* allele is a null in the absence of Cre and expresses *Brs3* once recombined.

**Figure 7:**
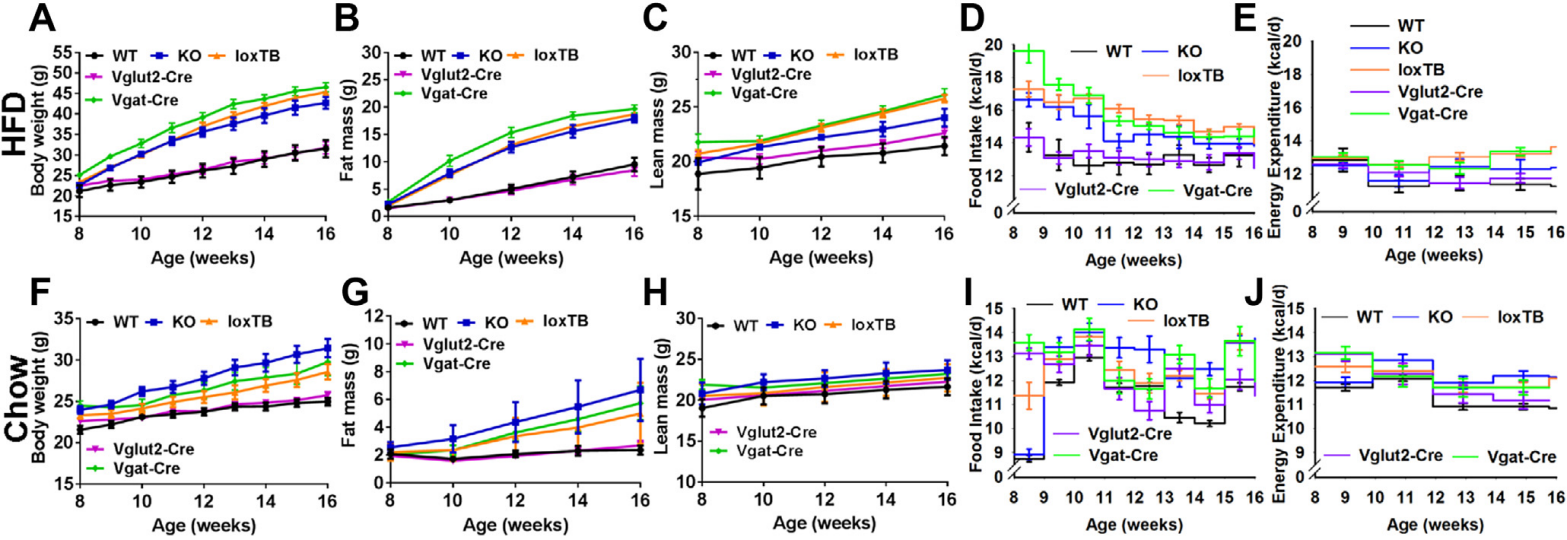
Effect of *Brs3* re-expression on body weight, composition, and energy homeostasis. *Brs3* was re-expressed in *Vglut2-* or *Vgat*-expressing neurons in mice fed a chow or high fat diet (HFD). (A–E) Body weight, fat mass, lean mass, food intake, and energy expenditure on a high fat diet (HFD) of WT, *Brs3*^*-/y*^ (KO), *Brs3*^*loxTB/y*^ (loxTB), *Brs3*^*loxTB/y*^*;Vglut2-Cre* (Vglut2-Cre), and *Brs3*^*loxTB/y*^*;Vgat-Cre* (Vgat-Cre) mice (N = 5 WT, 5 KO, 19 loxTB, 10 Vglut2-Cre, 6 Vgat-Cre). (F–J) as above, on a chow diet (N = 8 WT, 8 KO, 16 loxTB, 10 Vglut2-Cre, 6 Vgat-Cre). P values were determined by 2-way RM ANOVA and are in Table S2.

**Figure 8:**
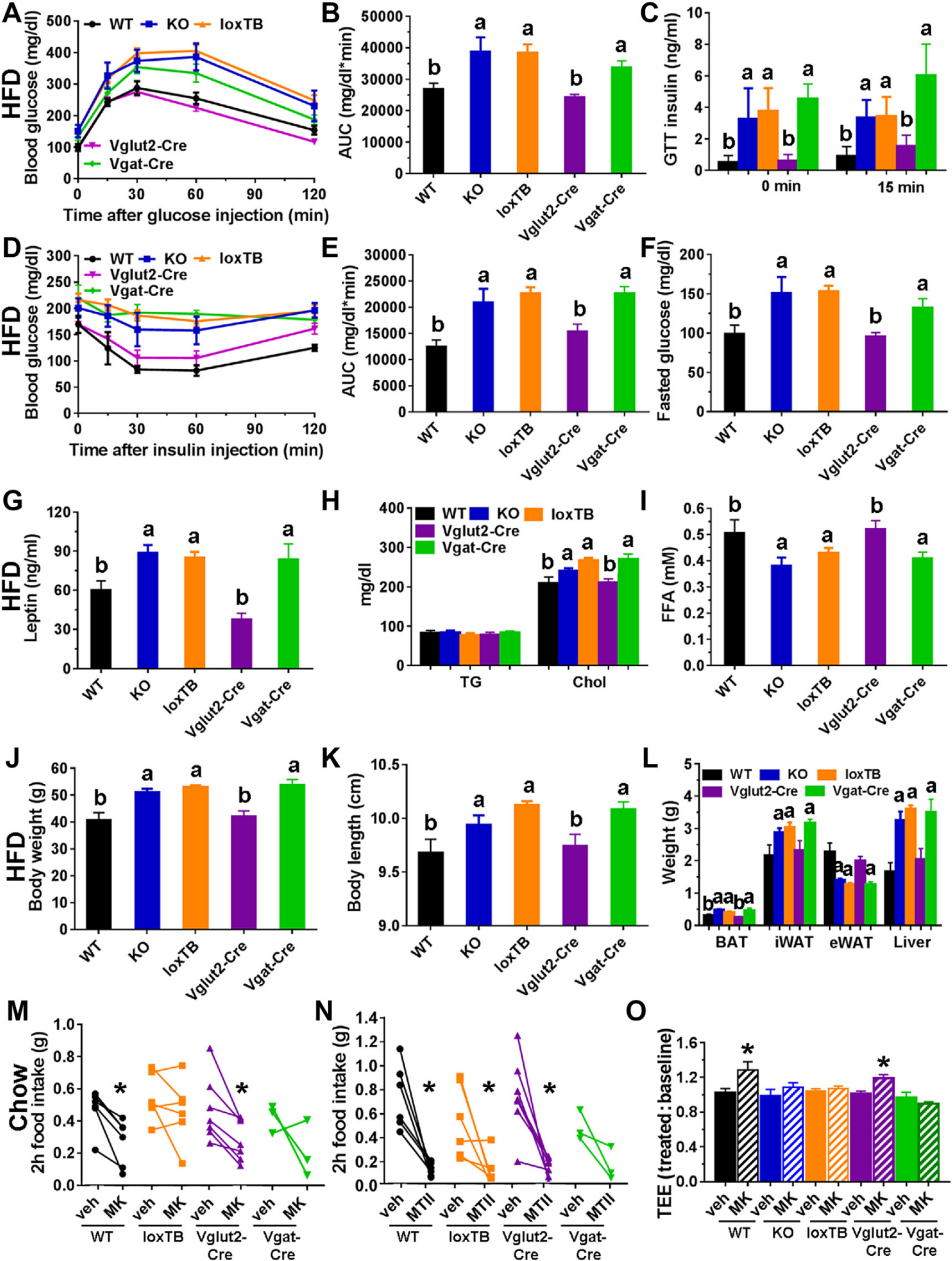
Phenotypes of mice with *Brs3* re-expression in *Vglut2-* or *Vgat-*expressing neurons. Groups are: WT, *Brs3*^*-/y*^ (KO), *Brs3*^*loxTB/y*^ (loxTB), *Brs3*^*loxTB/y*^*;Vglut2-Cre* (Vglut2-Cre), and *Brs3*^*loxTB/y*^*;Vgat-Cre* (Vgat-Cre) mice. Mice in A–L were fed a HFD and M–O were on chow. (A–C) Glucose tolerance test, area under the curve (AUC) of glucose tolerance test, and insulin at 0 and 15 min after glucose challenge. (D, E) Insulin tolerance test and area under the curve (AUC) of insulin tolerance test. (F) Fasting blood glucose. (G–I) Serum leptin, triglyceride (TG), cholesterol, and free fatty acid (FFA) levels. (J–L) Body weight, length, and tissue weights at 25 weeks (N = 5 WT, 5 KO, 19 loxTB, 10 Vglut2-Cre, 6 Vgat-Cre). Levels not sharing a letter are different at P < 0.05 determined by 1-way ANOVA with Holm-Sidak post-hoc testing. (M–N) Two-hour food intake after treatment with vehicle or MK-5046 (10 mg/kg, i.p) or MTII (3 mg/kg, i.p.) (N = 3–7/group). (O) Effect of MK-5046 (10 mg/kg, i.p.) on energy expenditure (N = 4–7/group). * indicates P < 0.05, drug vs vehicle, within genotype.

On a HFD, reference *Brs3*^*-/y*^ KO mice were obese compared to WT mice with increased food intake and greater body weight and adiposity (Figure 7A–E). The *Brs3*^*loxTB/y*^ mice were similarly obese and hyperphagic as the reference *Brs3*^*-/y*^ mice, confirming that unrecombined *Brs3*^*loxTB*^ is a null allele. The re-expression of *Brs3* in *Brs3*^*loxTB/y*^*;Vglut2-Cre* mice prevented the null phenotype of null mice, rescuing the body weight, fat mass, lean mass, food intake, and energy expenditure to levels that were remarkably similar to the WT mice. In contrast, re-expression of *Brs3* in *Brs3*^*loxTB/y*^*;Vgat-Cre* mice had no effect, with the body weight, fat mass, and lean mass being similar to that of null (both *Brs3*^*-/y*^ and *Brs3*^*loxTB/y*^) mice. Consistent with the adiposity changes, *Brs3*^*-/y*^, *Brs3*^*loxTB/y*^ and *Brs3*^*loxTB/y*^*;Vgat-Cre* mice had heavier BAT, iWAT and liver, longer body length, although they had decreased eWAT, compared to WT and *Brs3*^*loxTB/y*^*;Vglut2-Cre* mice (Figure 8J–L).

Glucose homeostasis was evaluated in the HFD cohort. In each case the more obese mice (*Brs3*^*-/y*^, *Brs3*^*loxTB/y*^, and *Brs3*^*loxTB/y*^*;Vgat-Cre*) had similar values, which were different from the leaner mice (WT and *Brs3*^*loxTB/y*^*;Vglut2-Cre*). The obese mice had higher fasting glucose and insulin levels, glucose intolerance, and insulin resistance by insulin tolerance test (Figure 8A–F). The obese mice had higher leptin and cholesterol and lower free fatty acids (Figure 8G–I).

On a chow diet, the phenotype was milder than in the HFD cohort, with a smaller increase in body weight and fat mass, and food intake and metabolic rate (Figure 7F–J). Measures of glucose homeostasis were consistent with the adiposity changes, with the relatively obese groups (*Brs3*^*-/y*^, *Brs3*^*loxTB/y*^ and *Brs3*^*loxTB/y*^*;Vgat-Cre*) having a tendency to impaired glucose and insulin tolerance and increased serum leptin levels, compared to the leaner groups (WT and *Brs3*^*loxTB/y*^*;Vglut2-Cre*). No consistent changes in serum TG, cholesterol, and FFA were observed (Figure S3).

The reduction in food intake caused by MK-5046 was similar to that in WT mice in *Brs3*^*loxTB/y*^*;Vglut2-Cre*, but lost in *Brs3*^*loxTB/y*^ mice (Figure 8M). Suppression of food intake by melanocortin agonist MTII was intact in all mice, including *Brs3*^*loxTB/y*^ mice (Figure 8N). MK-5046 increased energy expenditure in WT and *Brs3*^*loxTB/y*^*;Vglut2-Cre*, but not *Brs3*^*-/y*^, *Brs3*^*loxTB/y*^, or *Brs3*^*loxTB/y*^*;Vgat-Cre* mice (Figure 8O) [26].

## 4 Discussion

Determining the functions of *Brs3* in mammals has been hampered by the lack of an identified endogenous ligand. This orphan status precludes the usual paradigm for unraveling a receptor's physiology—historically, having a hormone/ligand paved the way to identification of its cognate receptor, which often occurred after much of the biology was understood. In contrast, most of our knowledge to date about *Brs3* has been acquired from genetics, by studying global KO mice. To refine the genetic approach, we developed mice that allow Cre-dependent selective ablation or re-expression of *Brs3*. Using these tools, we find that *Brs3* expression in *Vglut2* neurons is both necessary and sufficient for the identified functions of *Brs3*.

Our observations focus on *Brs3* regulation of metabolic rate, body temperature, food intake, and body weight. We also attempted to study heart rate because resting heart rate is lower in global *Brs3* KO mice [27]. Indeed, with 4–6 mice/group, there was a hint that the heart rate effect is also mediated via *Brs3* in *Vglut2* neurons. However, these experiments are underpowered, so formally this remains unaddressed. Behavioral phenotypes have been reported for global *Brs3* KO mice [13], [42]. In our assays, the behavioral alterations of the global null mice were not sufficiently robust and reproducible for us to undertake behavioral characterization of the selective mutant mice. We also did not explore the regulation of glucose-dependent insulin secretion by *Brs3*, an action at the beta cell of the pancreatic islets [20]. A caveat is that our studies are loss of function or reconstitution of function, which are insensitive to redundant *Brs3* functions. Studies combining BRS-3 agonist treatment with genetics could uncover additional functions of *Brs3*. Overall, the results are reassuringly concordant with prior phenotyping of the global null. One surprise was the lack of obesity in singly-housed, chow-fed *Brs3*^*fl/y*^*;Vglut2-Cre* mice. These mice did have a reduced metabolic rate but also ate less and, thus, did not become obese. Note that we would not have identified a phenotype in these mice without measuring long-term food intake. Interestingly, a cohort of group-housed, chow-fed *Brs3*^*fl/y*^*;Vglut2-Cre* mice did become obese (32.2 ± 1.4 g vs 26.4 ± 0.7 g at 13 weeks). It is possible that single vs group housing is the relevant difference, but we have not pursued this observation further.

*Brs3* mRNA and/or immunoreactivity is present in the CNS, but it is reported that BRS-3 is also found in peripheral sites, including muscle, islet beta cells, testis, ovary, uterus, lung, and cancers including carcinoid/neuroendocrine [5], [20], [21], [22] (https://gtexportal.org/home/gene/BRS3). The observation that *Brs3* expression in *Vglut2* neurons is sufficient to reverse the global null phenotype, demonstrates that *Brs3* in this subset of neurons mediates the non-redundant activities of *Brs3*.

Developing drug treatments for obesity has proven to be a very difficult task. Multiple nuclei in the brain integrate peripheral and central signals and send messages elsewhere in the brain and peripherally to regulate energy homeostasis. Because receptors often have different functions in different brain regions, even if complete molecular target selectivity is achieved with a drug, multiple drug effects are likely. To avoid this problem, there has been a hope that BRS-3 agonists might have anti-obesity effects via pharmacologic actions outside of the brain, avoiding some side effects [28], [31], [43]. However, our results strongly indicate that BRS-3 agonists achieve anti-obesity efficacy via activating glutamatergic neurons, likely chiefly in the brain.

A potential confounder of our experiments is that Cre activity during development will cause gene deletion, even if the Cre is not present in the mature cells [44]. While we cannot completely rule this out, the complementarity of the deletion and re-expression phenotypes and the measured DNA deletion efficiencies indicate a role for *Brs3* in mature *Vglut2* neurons. Note that *Brs3* also appears to be expressed in non-glutamatergic hypothalamic neurons, including GABAergic cells, because *Brs3* mRNA is only partially reduced in *Brs3*^*fl/y*^*;Vglut2-Cre* mice and re-expressed in *Brs3*^*loxTB/y*^*;Vgat-Cre* mice. The functions of *Brs3* in the non-glutamatergic cells remain to be determined.

We have identified *Brs3* neurons that express *Vglut2* as functionally important in energy homeostasis. While many neurons express *Vglut2*, relatively few express *Brs3*. *Brs3*-expressing neurons are located in, among other brain regions, the preoptic area (POA), paraventricular nucleus of the hypothalamus (PVH), dorsomedial hypothalamus (DMH), medial amygdala, and parabrachial nucleus (PBN) [12], [13], [14], [15], [16], [17], [18], [19]. Using in situ hybridization, Zhang et al. reported that many *Brs3* neurons are glutamatergic, while few are GABAergic [17]. We (unpublished observations) and others [41] have also found *Brs3* expression in GABAergic neurons. The RT-PCR data indicates that *Brs3* is expressed in both *Vglut2* and *Vgat* neurons in the hypothalamus. *Brs3* may also have functions in *Vgat* neurons, with a slight reduction in body weight on chow diet and slightly impaired glucose tolerance on HFD in *Brs3*^*fl/y*^*;Vgat-Cre* mice.

We have speculated that low levels of a BRS-3 endogenous agonist signals an energy deficit, either acute or chronic (possibly analogous to cholecystokinin or leptin, respectively). Thus BRS-3 ligand deficiency signals an underfed state and evokes the physiological responses appropriate to that state [29]. A next research goal is elucidation of the specific glutamatergic *Brs3* neurons responsible for regulation of food intake, metabolic rate, body temperature, and adiposity. Nuclei expressing *Brs3* and also having glutamatergic neurons that inhibit food intake include the PVH [45], PBN [46], and lateral hypothalamus [47]. Glutamatergic neurons that activate BAT and increase energy expenditure and Tb are located in the DMH, PVH, and PBN [48], [49], [50]. In the POA, the best-studied thermoregulatory glutamatergic neurons decrease Tb but do not increase it [41], [51]. Functional experiments, such as with optogenetics and chemogenetics, are required to further localize the specific nuclei and circuits where *Brs3* regulates the different aspects of energy homeostasis.
