# Corrigendum to “Bombesin-like receptor 3 (*Brs3*) expression in glutamatergic, but not GABAergic, neurons is required for regulation of energy metabolism” [Mol Metabol 6 (2017) 1540–1550]

**DOI:** 10.1016/j.molmet.2017.12.009

**Published:** 2017-12-27

**Authors:** Cuiying Xiao, Ramón A. Piñol, Jesse Lea Carlin, Cuiling Li, Chuxia Deng, Oksana Gavrilova, Marc L. Reitman

**Affiliations:** 1 Diabetes, Endocrinology, and Obesity Branch, National Institute of Diabetes and Digestive and Kidney Diseases, NIH, Bethesda, MD 20892, USA; 2 Genetics of Development and Disease Branch, National Institute of Diabetes and Digestive and Kidney Diseases, NIH, Bethesda, MD 20892, USA; 3 Faculty of Health Sciences, University of Macau, Macau SAR, China; 4 Mouse Metabolism Core, National Institute of Diabetes and Digestive and Kidney Diseases, NIH, Bethesda, MD 20892, USA

In the original article, the PCR genotyping protocol contained errors. The correct x214 primer sequence is 5′-TTTCTCTGGGAATGACAATGC. The correct PCR product sizes are 191 bp for the wild type allele and 249 bp for the floxed allele. These corrections do not affect the conclusions of the paper, and the authors sincerely apologize for this oversight.

*Generation of Brs3 conditional allele, Brs3^fl^* having the floxed targeted allele with the selection cassette removed was selected. PCR genotyping used primers x213 (5′-GTATGCAT TACCACGTACGA, intron 1, forward) and x214 (5′-TTTCTCTGGGAATGACAATGC, intron 1, reverse), producing products of 191 bp wild type (WT) and 249 bp (floxed). Brs3 mRNA was quantitated by RT-PCR using primers x573 (5′-CTGCTGACTTGTGTGCCTGT) and x574 (5′-AGTGGCTTCACGACTGCTTT).

