# Supplementary material for: Bombesin-like receptor 3 (Brs3) expression in glutamatergic, but not GABAergic, neurons is required for regulation of energy metabolism
Source: Mol Metab. 2017 Sep 15;6(11):1540–50. doi: 10.1016/j.molmet.2017.08.013 (PMC5681273; doi:10.1016/j.molmet.2017.08.013)
Supplement: Supplementary file 1 [file mmc1.pdf]

**Table S1. Effect of genotype on body temperature**

| | N | Tb mean (°C) | Tb SD (°C) | Tb 5th percentile (°C) | Tb 95th percentile (°C) | Tb range (°C) |
|---|---|---|---|---|---|---|
| WT | 10 | 36.97 ±0.17 | 0.778 ±0.031 | 35.87 ±0.18 | 38.21 ±0.15 | 2.34 ±0.06 |
| *Brs3*$^{-/y}$ | 10 | 36.67 ±0.08 | 0.944 ±0.029 | 35.19 ±0.10 | 38.08 ±0.07 | 2.88 ±0.08 |
| *P* | | 0.14 | **0.0009** | **0.004** | 0.43 | **0.00008** |
| | | | | | | |
| *flox/y* | 11 | 35.96 ±0.05 | 0.837 ±0.039 | 34.79 ±0.09 | 37.46 ±0.08 | 2.66 ±0.11 |
| *Vglut2-Cre* | 12 | 35.92 ±0.07 | 1.055 ±0.037 | 34.40 ±0.11 | 37.65 ±0.07 | 3.25 ±0.10 |
| *P* | | 0.62 | **0.0006** | **0.01** | 0.09 | **0.0008** |
| | | | | | | |
| *flox/y* | 10 | 36.05 ±0.08 | 0.813 ±0.026 | 34.88 ±0.08 | 37.39 ±0.08 | 2.51 ±0.07 |
| *Vgat-Cre* | 12 | 36.03 ±0.06 | 0.793 ±0.036 | 34.89 ±0.06 | 37.37 ±0.09 | 2.48 ±0.09 |
| *P* | | 0.83 | 0.67 | 0.95 | 0.85 | 0.79 |

Body temperature (Tb) was measured by telemetry for 24 hour periods and analyzed as described in Methods. The Tb SD is the standard deviation of the ~1440 observations during the 24 hours. The Tb range is the difference between the 95$^{th}$ and 5$^{th}$ percentiles. The first set (WT and *Brs3*$^{-/y}$) is a reanalysis of data from [26]. Paired sets were acquired concurrently, data are mean ±standard error, with *P* values determined by t-test.

**Table S2.**
**P values for Figure 7, Effect of *Brs3* re-expression on body weight, composition, and energy homeostasis.**

| Panel: | A | B | C | D | E | F | G | H | I | J |
|---|---|---|---|---|---|---|---|---|---|---|
| | **DIO BW** | **DIO fat** | **DIO lean** | **DIO FI** | **DIO EE** | **chow BW** | **chow fat** | **chow lean** | **chow FI** | **chow EE** |
| **WT vs KO** | <0.001 | <0.001 | 0.24 | 0.032 | 0.80 | 0.002 | <0.001 | 0.040 | 0.010 | 0.12 |
| **WT vs loxTB** | <0.001 | <0.001 | 0.002 | <0.001 | 0.031 | 0.14 | 0.056 | 0.38 | 0.12 | 0.15 |
| **WT vs Vgat-Cre** | <0.001 | <0.001 | 0.004 | <0.001 | 0.18 | 0.091 | 0.054 | 0.16 | 0.25 | 0.33 |
| **WT vs Vglut2-Cre** | 0.73 | 0.89 | 0.54 | 0.95 | 0.82 | 0.67 | 0.98 | 0.77 | 0.72 | 0.42 |
| **KO vs loxTB** | 0.64 | 0.73 | 0.50 | 0.22 | 0.35 | 0.12 | 0.11 | 0.45 | 0.50 | 0.99 |
| **KO vs Vgat-Cre** | 0.22 | 0.18 | 0.44 | 0.24 | 0.68 | 0.60 | 0.46 | 0.71 | 0.59 | 0.98 |
| **KO vs Vglut2-Cre** | <0.001 | <0.001 | 0.45 | 0.025 | 0.92 | 0.004 | <0.001 | 0.18 | 0.041 | 0.98 |
| **loxTB vs Vgat-Cre** | 0.31 | 0.16 | 0.56 | 0.84 | 0.75 | 0.58 | 0.83 | 0.67 | 0.92 | 0.87 |
| **loxTB vs Vglut2-Cre** | <0.001 | <0.001 | 0.010 | <0.001 | 0.026 | 0.23 | 0.040 | 0.65 | 0.36 | 0.98 |
| **Vgat-Cre vs Vglut2-Cre** | <0.001 | <0.001 | 0.022 | <0.001 | 0.25 | 0.13 | 0.052 | 0.42 | 0.43 | 1.00 |

2-Way RM ANOVA with post testing (Holm-Sidak method)

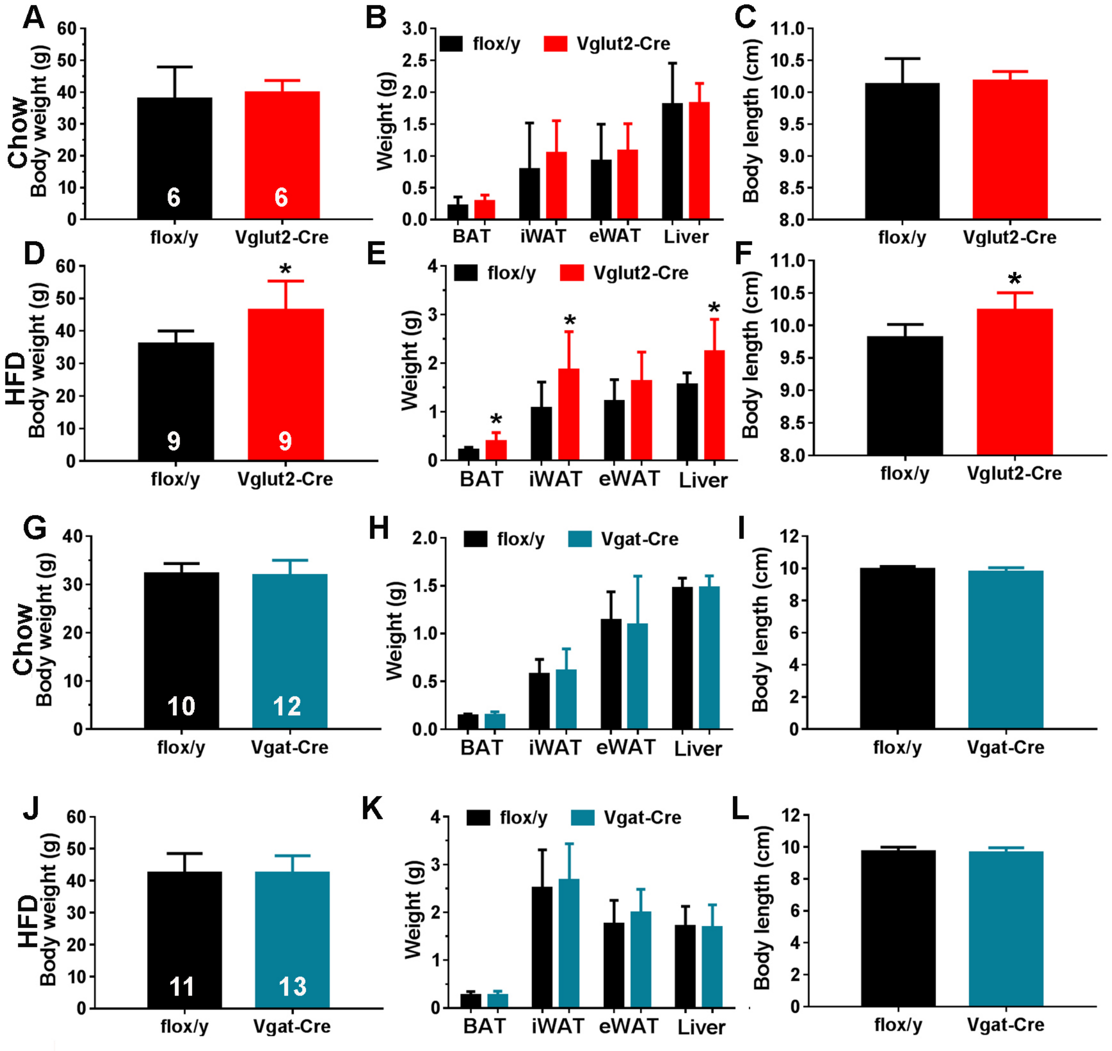


**Figure S1**. **Body and tissue weights of mice with *Brs3* deletion in *Vglut2*- or *Vgat*-expressing neurons**. *Brs3* was deleted in *Vglut2*- or *Vgat*-expressing neurons in mice fed a chow or high fat diet (HFD). (A-C) *Brs3*$^{fl/y}$;*Vglut2-Cre* (Vglut2-Cre) and littermate control *Brs3*$^{fl/y}$ (flox/y) mice on chow at 53 weeks. (D-F) the same genotypes on HFD at 51 weeks. (G-I) *Brs3*$^{fl/y}$;*Vgat-Cre* (Vgat-Cre) and littermate control *Brs3*$^{fl/y}$ (flox/y) mice on chow at 52 weeks. (J-L) the same genotypes on HFD at 25 weeks. *, P<0.05 by *t*-test.

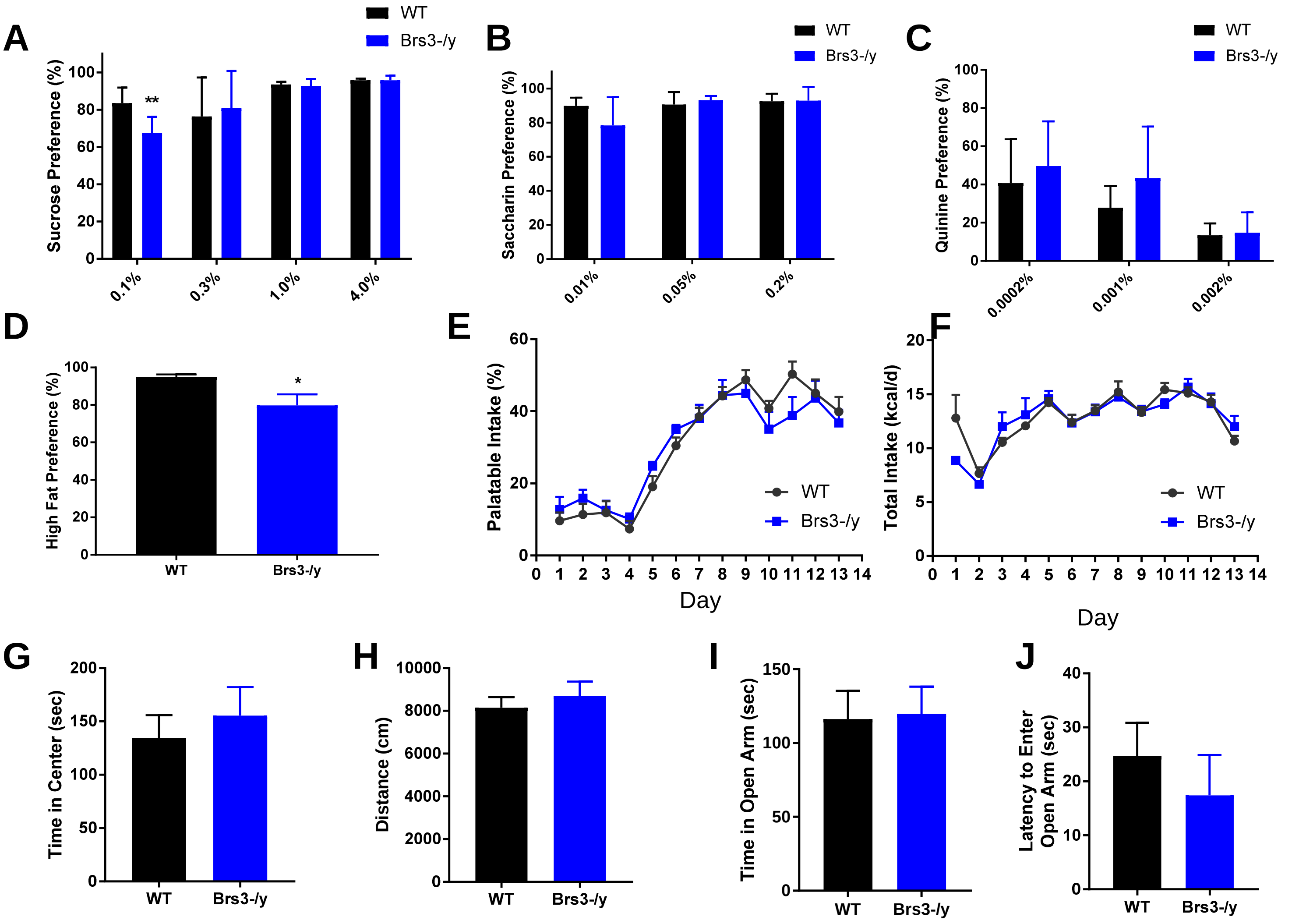


**Figure S2. Behavior phenotypes in *Brs3*$^{-/y}$ mice.** (A-C) Drinking preference for water vs sucrose, saccharin, or quinine solution. Mice were individually housed in standard cages for four days with ad libitum access to chow, tap water, and test solution (sucrose, saccharin, or quinine at the indicated concentration, w/v, in separate experiments). Water and test bottle positions were reversed daily. Preference was calculated using the average consumed on days 3 and 4: preference % = 100 x (test solution consumed)/(test solution consumed + water consumed). (D) Preference for high fat vs chow diet. A similar design was used, with ad libitum access to chow and high fat diet (60% kcal fat, # D12492, Research Diets Inc) for four days, with consumption measured in g on the last two days. (E-F) One-hour palatable food intake. Mice were individually housed for 14 days with ad libitum access to chow. Each day at noon (lights on: 0600-1800), the mice were given access to either palatable high-fat/high-sugar food (Reese's Peanut Butter chips) or chow for one hour. (E) Chow and palatable food intake was measured daily and expressed as: preference %= [(1 h palatable food)/(1 h palatable food + 24 h chow intake) × 100] using kcal as units. (F) Daily total caloric intake. (G-H) Open field test. Mice were placed in the middle of an open field (60cm x 60cm). (G) Time in the center (20cm x 20cm) area and (H) distance traveled were measured for 30 min by video tracking software (Ethovision X10, Noldus). (I-J) Elevated zero maze. Mice were placed into a closed arm of an elevated zero maze and (I) time spent in open arms and (J) latency to enter open arms were measured for 10 min with video tracking software. Data are mean ± SEM, n = 7-8/group. * $p < 0.05$, ** $p < 0.01$ using t-test.

Behavior tests were performed in *Brs3*$^{-/y}$ and WT littermates at: sucrose, 14-18 weeks (WT 27.6 g, *Brs3*$^{-/y}$ 30.0 g), saccharine, 19-21 weeks (WT 29.7 g, *Brs3*$^{-/y}$ 34.9 g), quinine, 23-26 weeks (WT 30.7 g, *Brs3*$^{-/y}$ 36.8 g), and high fat 31 weeks (WT 34.6 g, *Brs3*$^{-/y}$ 43.7 g). Open field and zero maze used a different cohort at 20-22 weeks old (WT 27.2 g, *Brs3*$^{-/y}$ 31.9 g). The sucrose, quinine, and high fat preference assays and open field test were repeated in an independent cohort with similar findings. The repeat 0.1% sucrose preference test did not find a significant difference between genotypes. In the high fat preference test, *Brs3*$^{-/y}$ mice again had a lower preference for high fat diet (WT 96.1 ±1.6 %, *Brs3*$^{-/y}$ 85.8 ±4.2 %, p=0.049).

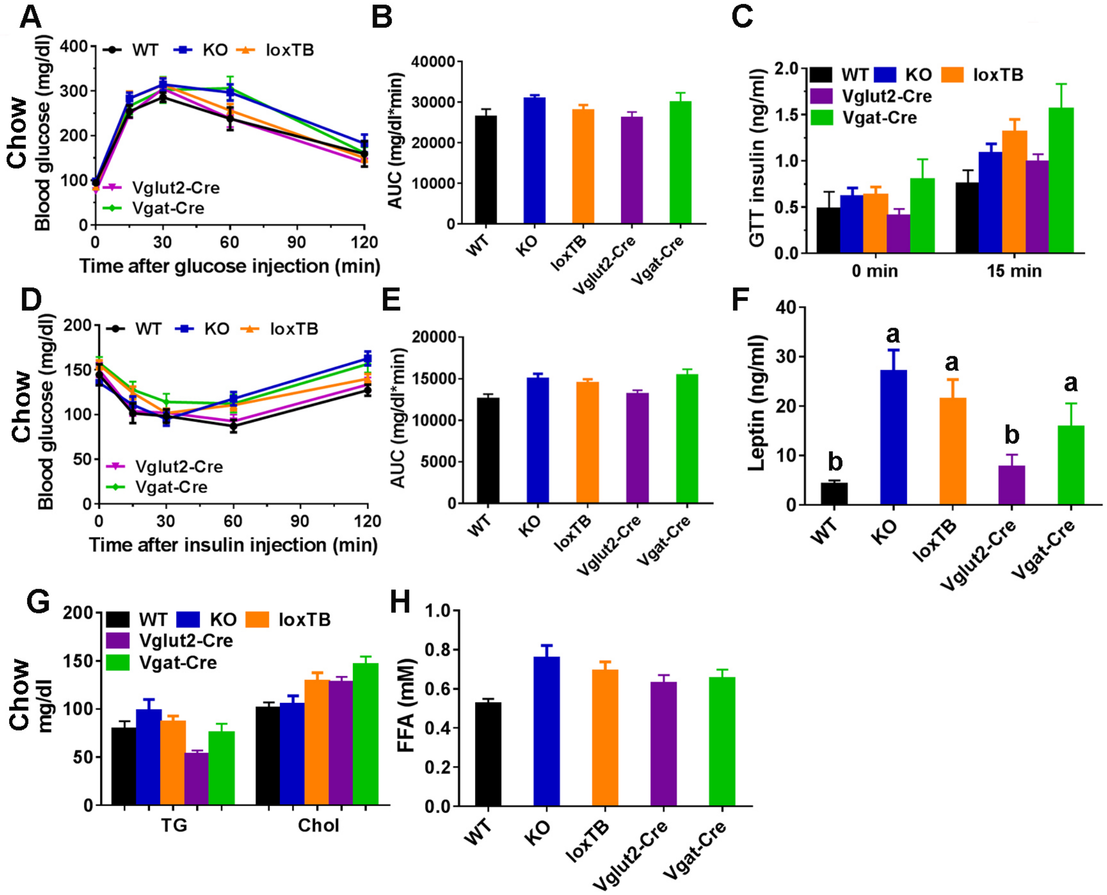


**Figure S3**. **Glucose and lipid metabolism after *Brs3* re-expression in *Vglut2*- or *Vgat*-expressing neurons in mice fed chow**. Groups are: WT, global Brs3 knockout (KO), *Brs3*$^{loxTB/y}$ (loxTB), *Brs3*$^{loxTB/y}$*;Vglut2-Cre* (Vglut2-Cre), and *Brs3*$^{loxTB/y}$*;Vgat-Cre* (Vgat-Cre) mice. (A-C) Glucose tolerance test, area under the curve (AUC), and insulin at indicated times after glucose challenge. (D,E) Insulin tolerance test and AUC. (F) Serum leptin levels. (G,H) plasma triglyceride (TG), cholesterol, and free fatty acid (FFA) levels. N=8 WT, 8 KO, 16 loxTB, 10 Vglut2-Cre, 6 Vgat-Cre. Levels not connected by same letter are different ($P<0.05$) by 1-way ANOVA with Holm-Sidak post-hoc testing.
